# Microstructure Evolution of AlSi10Mg(Cu) Alloy Related to Isothermal Exposure

**DOI:** 10.3390/ma11050809

**Published:** 2018-05-16

**Authors:** Cheng Cai, Huifang Geng, Shifu Wang, Boxue Gong, Zheng Zhang

**Affiliations:** 1 Key Laboratory of Aerospace Materials and Performance (Ministry of Education), School of Materials Science and Engineering, Beijing University of Aeronautics and Astronautics, Beijing 100191, China; buaamsecc@163.com (C.C.); wwangshifu@163.com (S.W.); bzscofield@buaa.edu.cn (B.G.); 2 The Collaborative Innovation Center for Advanced Aero-Engine (CICAAE), Beijing University of Aeronautics and Astronautics, Beijing 100191, China; 3 Beijing Key Laboratory of Advanced Nuclear Materials and Physics, Beijing University of Aeronautics and Astronautics, Beijing 100191, China; 4 ELI-ALPS, ELI-HU Non-Profit Limited, Dugonics ter 13, Szeged 6720, Hungary

**Keywords:** isothermal exposure, microstructure, mechanical properties, corrosion resistance, microstructure evolution

## Abstract

The mechanical properties and corrosion resistance changes of AlSi10Mg(Cu) alloy under different isothermal exposure conditions have been investigated by tensile experiments and electrochemical testing. The results show that isothermal exposure has a significant influence on the mechanical properties and corrosion resistance. Tensile strength is more sensitive to the higher exposure temperature, while the corrosion resistance is greater affected by the lower exposure temperature and shorter time. Microstructure evolution of AlSi10Mg(Cu) alloy related to different isothermal exposure condition has also been studied by using transmission electron microscopy (TEM). The results indicate that the isothermal exposure changed the type and density of nanoscale precipitates in the alloy, which in turn induced the change of performance of the alloy.

## 1. Introduction

Due to their light weight and excellent mechanical properties aluminum alloys have been widely used in the automobile industries in the past few decades, for parts such as wheels and engines, etc. [1,2,3]. Properties of Al-Si-Mg alloys depends on the alloy compositions [4], casting process and the subsequent heat treatment process [5]. The good performance can be obtained by optimizing the volume fraction and the morphology of the main phase components (α-Al matrix and silicon crystals) and the secondary phases (β-AlFeSi, α-AlFeSiMg, Mg_2_Si and Q, etc.) [6].

In the past, mechanical properties of Al-Si-Mg alloys were intensively investigated by many researchers [5,7,8]. Jamaati et al. [7] and Suárez-Peña et al. [8] found that decreasing the amount of the eutectic phase or alter its morphology by quenching or addition of modifiers such as sodium or strontium can improve the final mechanical properties. Sjölander et al. [5] reviewed the effect of the heat treatment process on the mechanical properties, and suggested that the best alloy mechanical properties need to consider the entire heat treatment process rather than just the solution treatment and artificial aging parameters. From the corrosion point of view, the effect of the main phase components, such as eutectic Si and the secondary phase (Fe-containing phase and Mg_2_Si and Q, etc.) in the alloy on the corrosion behavior has also been extensively investigated [9,10]. Jain [9] found that Si and Fe-containing phase (β-AlFeSi or α-AlFeSiMg) are cathodic with respect to the Al matrix, which leads to the formation of microgalvanic couples. Many researchers [10,11,12,13] studied the effect of ageing on the main phase of the aluminum alloy. Yasakau et al. [10] investigated the role of intermetallic phases in localized corrosion of AA5083 by in-situ atomic force microscopy. They found that the Mg_2_Si phase has a potential lower relative to the Al-matrix, which may enhance location corrosion. Ding et al. [11] studied the microstructure evolution behavior of A356.2 alloy under cyclic thermal exposure conditions, and they found eutectic silicon increased with exposure temperature and exposure time increasing. However, Zhou et al. [12] conducted a comparative study of the damage behavior of eutectic Si phase in AlSi10Mg(Cu) alloy under low-cycle fatigue and thermal fatigue conditions by the in-situ observation method; they found the silicon morphology of the alloy did not change. Besides, the morphology of iron intermetallic particles in cast aluminum are not substantially altered during heat treatment (below 540 °C), which depended on both the concentration of elements (Fe, Si, Mg, Mn, etc.) and cool rate [13].

The modern engine cylinder head, one of the most important components of an automobile engine, has to continuously work at a high temperature (over 250 °C at the bridge) for a long period of time. The mechanical properties of a heat treatable aluminum alloy depend on the type and density of the strengthening phase, which is affected by the heat treatment history [14]. Izcara and coworkers [15] confirmed that as the aging temperature and time increase, the size distribution of the strengthening precipitates increases, while the density of the strengthening precipitates decreases. Sjölander et al. [16] and Ibrahim et al. [17] investigated the effects of ageing conditions on the mechanical properties of Al-Si-Mg alloy. They found that the mechanical properties of Al-Si-Mg alloy depended on the ageing conditions. Intergranular corrosion behavior of aluminum alloy 7150 were investigated by Ramgopal et al. [18]. They found that corrosion behavior of 7150 alloy depended on the tempers parameters (such as temperature and time).

Despite numerous investigations invested into the properties of Al-Si-Mg alloy, the microstructure evolution of AlSi10Mg(Cu) alloy related to the isothermal exposure is very limited. In this study, the effect of isothermal exposure on the microstructure evolution of AlSi10Mg(Cu) alloy was examined by TEM. Meantime, interaction mechanisms among the exposure process, microstructure, tensile strength and corrosion resistance are discussed in detail.

## 2. Materials and Experiments

**Figure 1:**
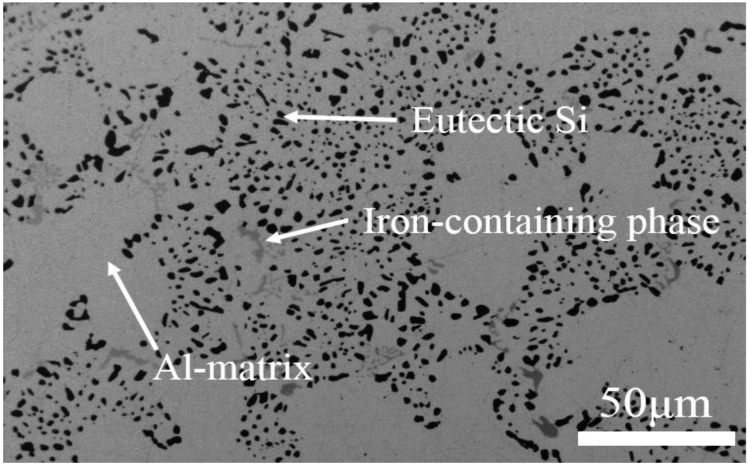
Typical scanning electron microscope (SEM) image showing the microstructure of as-received AlSi10Mg(Cu) alloy.

The used experimental material is AlSi10Mg(Cu) alloy drawn directly from the cylinder head, and its chemical compositions (wt %) are 10.33 Si, 0.40 Mg, 0.36 Fe, 0.28 Cu, 0.32 Mn, 0.16 Zn, 0.10 Ti and balance Al. The typical microstructure of as-received alloy contains eutectic silicon, aluminum matrix and a small amount of iron-containing phases (Figure 1). Before T6 ageing (180 °C for 4 h), materials were solution-treated at 535 °C for 6h followed by quenching in water at room temperature. Two different exposure temperatures (180 and 270 °C) and various times (0~90 h) were selected in this research. The specimen were heated to the selected temperature at a heating rate of 10 °C/min and held for various amounts of time (0~90 h) in the heat treatment furnace, followed by quenching in water to room temperature (RT) immediately.

Tensile samples were machined according to the ASTM E8M [19] with a rectangular cross-section of 5 mm × 2 mm and a gauge length of 15 mm. Room temperature tensile tests were performed on an Instron 8801 servo-hydraulic testing machine (Instron Corporation, Norwood, MA, USA) equipped with an extensometer in air at a displacement rate of 5 × 10^−1^ mm/min. At least three tensile samples were tested for each condition to confirm the reproducibility. Ultimate tensile strength (UST), yield strength (YS) and elongation to failure (A) were derived from the data acquisition system.

A CHI660B electrochemical testing instrument (CH Instruments, Inc., Shanghai, China) connected to a three-electrode cell was used for the potentiodynamic polarization experiments. The working electrode was the test material with an immersed area of 1 cm^2^. Saturated calomel electrode (SCE) was invoked as the reference electrode and Platinum was regarded as the auxiliary electrode. The potentiodynamic polarization experiments were performed under a scan rate of 1 mV/s. The scan was conducted range from −1.40 mV to −0.30 mV with respect to open-circuit potential. These experiments were carried out in 3.5 wt % NaCl solution at 25 °C. Noted that all the electrochemical experiments were performed in the environment chamber to ensure the same experimental temperature.

In order to track the microstructure evolution of the studied alloy during the isothermal exposure, the transmission electron microscope (TEM) observations were performed on the FEI Tecnai F20 microscopy (FEI Company, Hillsboro, OR, USA) operating at 300 kV. Samples for TEM examination were cut from the isothermal exposed specimens, and then ground into ~50 μm thin foils. Several disks with a diameter of 3 mm were punched from these thin foils, and subsequently prepared by dimpling disks in a Gantan 691 Precision Ion Polishing System with a small incident angle until perforation.

## 3. Results

### 3.1. The Evaluation of Mechanical Properties

**Figure 2:**
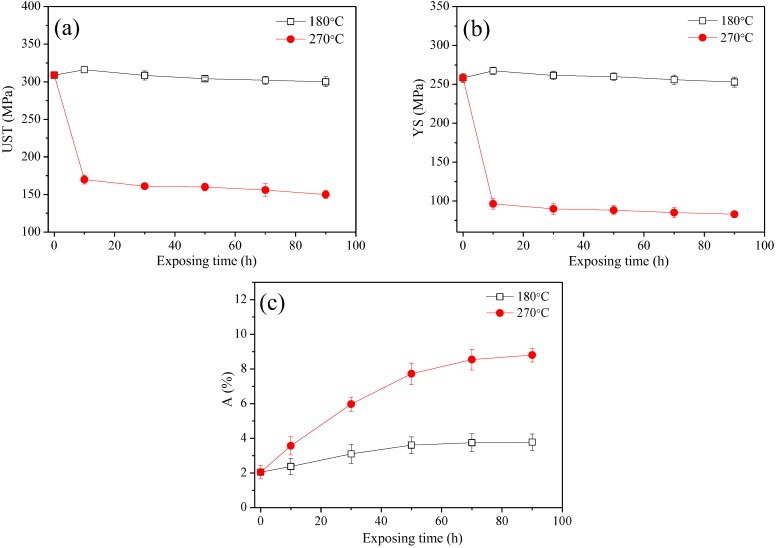
Tensile properties of the studied alloy versus exposure time: (**a**) UST, (**b**) YS and (**c**) A.

Figure 2 shows the engineering tensile properties of the studied alloy versus exposure time. At an exposure temperature of 180 °C. As shown in Figure 2a,b, UST and YS show the similar trend as a function of exposure time: The studied alloy reaches its peak strength after 10 h of exposure, and further exposure led to a slow decrease in strength. At the same time, A shows a continuous growth trend with increasing exposure time. It is interesting that the alloy simultaneously improves its strength and ductility after exposed at 180 °C after 10 h. A possible reason is that the alloy has attenuated performance in the stored procedure. At an exposure temperature of 270 °C, UST and YS show the also similar trend to exposure time: strength sharply decreases after 10 h of exposure and then remains stable. In contrast to strength, E of the studied alloy shows a steady increase.

**Figure 3:**
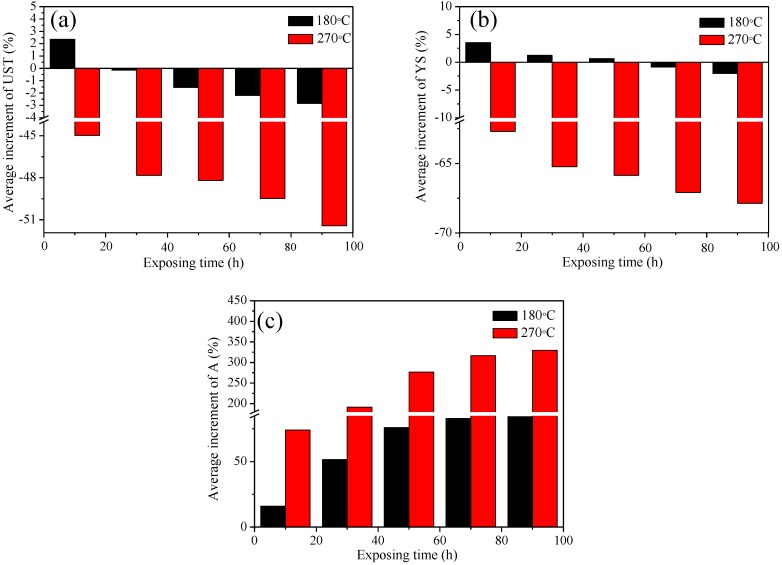
Average increment of tensile properties of the studied alloy as a function of the exposure time. (**a**) UST, (**b**) YS and (**c**) A.

Figure 3 indicates the average increment of tensile results of the studied alloy as a function of the exposure time. After thermal exposure at 180 °C, the increments of UST and YS show a similar trend as that of strength: the increment of the strength reaches its peak and then slowly reduces. While at 270 °C, tensile strength (UST and YS) of the studied alloy drastically decreases after 10 h of exposure, and then the strength of the alloy is subsequently maintained at a constant and no longer changes. Thermal exposure leads to a similar growth trend in A for the tested alloy at both the studied temperatures. However, the higher temperature leads to a more noticeable impact on the tensile result of the alloy. The as-received sample is selected as the reference sample, which shows the YS of ~258.23 MPa and an elongation to failure of ~2.39%. For example, after exposed at 270 °C for 90 h, the strength of the alloy is ~48.37% lower than that of the alloy exposed at 180 °C, while the A of the alloy is ~132.80% higher than that of the alloy exposed at 180 °C. Based on the tensile results, the as-received alloy and the alloy exposed for 90 h are selected as the representative to study their electrochemical performance to reveal the relationship between electrochemical performance and the exposure parameters.

**Figure 4:**
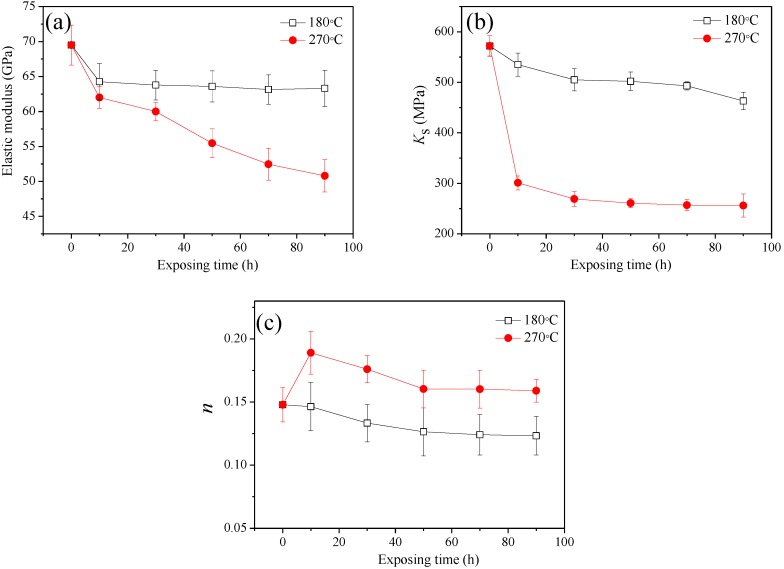
Parameters (elastic modulus, *K*_s_ and *n*) of the studied alloy vs exposing time: (**a**) elastic modulus, (**b**) *K_s_* and (**c**) *n*.

To better quantify the strain hardening behavior of the studied alloy, the stress–strain parameters for uniaxial loading were characterized using the Hollomon power law (S = *K_s_*·ε*^n^*). Parameters (elastic modulus, *K*_s_ and *n*) of the studied alloy under different conditions vs exposing time were shown in Figure 4. Obviously, modulus and strain hardening parameters (*K*_s_ and *n*) are temperature-sensitive. After heat exposure of more than 10 h, elastic modulus drop rapidly, and stabilize at 180 °C, but continue to decline at 270 °C (Figure 4a). This may be due to the low performance of the alloy after high temperature exposure. The strain-hardening coefficient as a function of exposing time was shown in Figure 4b. With the increase of exposure time, the strain hardening coefficient gradually decreases at 180 °C. However, under high temperature conditions (270 °C), *K_s_* rapidly declines to 52% of the as-received alloy after 10 h exposure, and then *K_s_* basically remains stable over time. The strain-hardening exponent *n* was evaluated for the uniform plastic deformation region between YS and UST point. The strain-hardening exponent as a function of exposure time was displayed in Figure 4c. It is remarkable that the studied alloy showed significantly higher strain hardening exponent at 270 °C than that at 180 °C. It indicates that the studied alloy has higher resistance to deformation after exposure at 270 °C than that at 180 °C. This phenomenon may, to a certain extent, be related to the transformation of the strengthening phase under high temperature conditions [20].

### 3.2. The Evaluation of Corrosion Resistance

**Figure 5:**
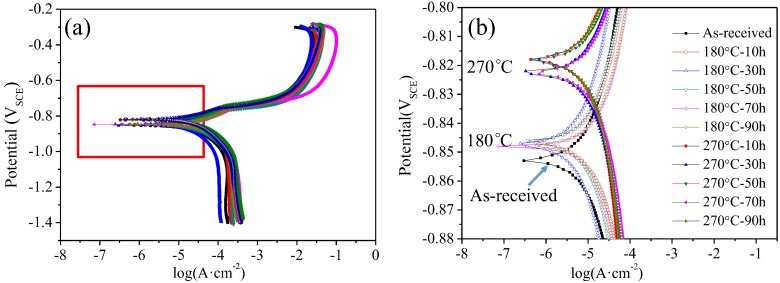
(**a**): Polarization curves of the studied AlSi10Mg(Cu) alloy under different temperatures and time in 3.5 wt % NaCl solution; (**b**) partial magnification of polarization curves marked by a red box in (**a**).

**Figure 6:**
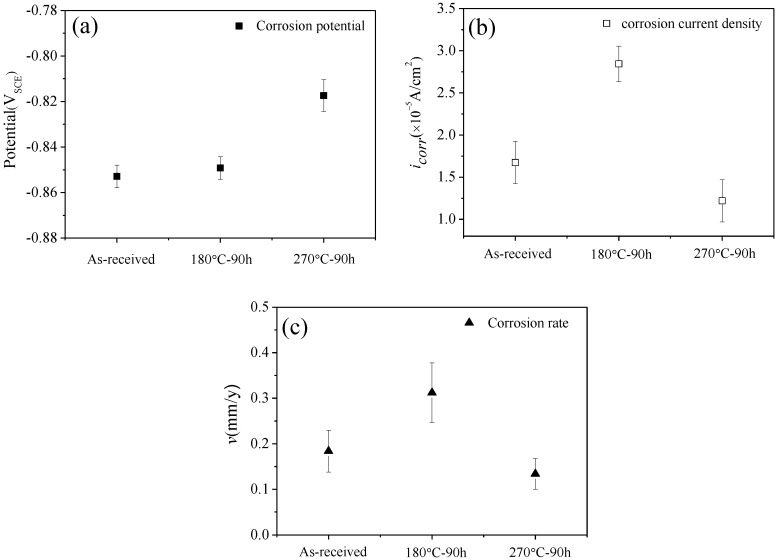
Effects of thermal exposure parameters on the corrosion properties of the studied alloy: (**a**) the corrosion potential; (**b**) the corrosion current density; (**c**) the corrosion rate.

Generally, the electrochemical impedance spectroscopy (EIS) tests can effectively evaluate the corrosion resistance of alloys by analyzing the corrosion reactions on working electrodes. In this section, the effect of the thermal exposure parameters on the corrosion resistance of AlSi10Mg(Cu) alloy was discussed by the results of EIS tests. Figure 5 shows the potentiondynamic polarization curves of the studied alloy under different thermal exposure parameters. The polarization curves obtained in different conditions have a similar shape, suggesting that the corrosion process subjected to different thermal exposure treatment has the same electrochemical mechanism. The electrochemical corrosion parameters derived from the polarization curves are shown in Figure 6. Here, the derived parameters are corrosion potential (*E*_corr_) and the corrosion current density (*i*_corr_), which are obtained based on Tafel extrapolation method [21,22]. Therefore, the corrosion potential and the current density of the as-received alloy can be evaluated as −0.853V_SCE_ and 1.674 × 10^−5^ A/cm^2^, respectively. From the results of the electrochemical tests, it can be seen that the electrochemical corrosion performance of the studied alloy is related to the exposure temperature and time. The influence of exposure temperature on the electrochemical corrosion parameters are as follows: From Figure 6a,b, the corrosion potential increases from −0.845 V_SCE_ to −0.819 V_SCE_, when the thermal exposure temperature is changed from 180 °C to 270 °C after 90 h. Meanwhile, the corrosion current density decreases from 2.839 × 10^−5^ A/cm^2^ to 1.219 × 10^−5^ A/cm^2^. Therefore, it can be concluded that the corrosion resistance of AlSi10Mg(Cu) alloy is affected by the exposure parameters. This is because the distribution of precipitates is affected by the exposure temperature and time [5,23].

The corrosion rate of the alloy is proportional to the corrosion current density, which can be calculated by [21,24]:

(1) $$v=K_{c}\frac{M}{n}\frac{1}{\rho }i_{\mathrm{corr}}$$

where $K_{c}$ is a conversion factor (3.27 × 10^−3^ mm/(μA·cm·y)); M is the molar mass of the metal (26.98 g/mol); n is the number of electrons exchanged in the dissolution reaction (3/mol); ρ is the density of the studied AlSi10Mg(Cu) alloy and its value is 2.68 g/cm^3^.

The corrosion rate obtained from the Equation (1) is shown in Figure 6c. Obviously, the corrosion rate of the studied alloy is considerably affected by exposure parameters. The relationship of microstructure and the corrosion resistance of AlSi10Mg(Cu) alloy will be discussed in Section 4.2.

### 3.3. Microstructures Observation

**Figure 7:**
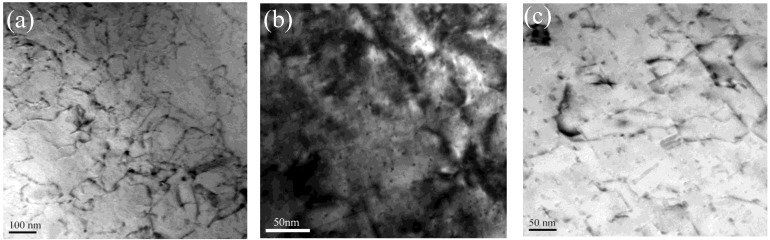
Bright field TEM images of the studied alloy suffered by different exposure conditions: (**a**) as-received; (**b**) 180 °C for 90 hl; (**c**) 270 °C for 90 h.

**Figure 8:**
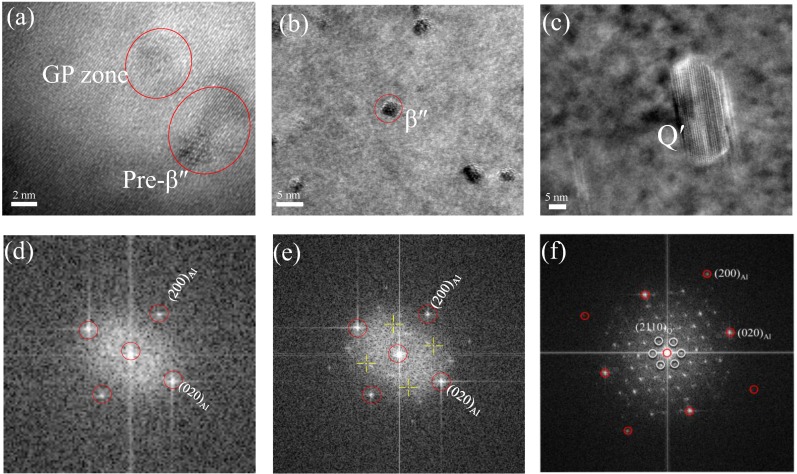
HRTEM images of the cross-section of the precipitates observed from <001>_Al_ in the different exposure conditions: (**a**) as-received, (**b**) 180 °C for 90 h, (**c**) 270 °C for 90 h, (**d**–**f**) corresponding FFT spectrums of precipitates in (**a**–**d**), respectively.

Figure 7 shows the bright-field TEM images of the studied alloy suffered by different exposure conditions. A typical microstructure obtained from the as-received alloy is illustrated in Figure 7a. At this stage, no clear precipitate was observed with the exception of the high-density dislocation distributed in the Al matrix. Figure 7b shows the microstructure of the sample exposed at 180 °C for 90 h. Lots of precipitates (around 2~5 nm in diameter) can be observed in the Al matrix. However, the dislocation density is less than that of the as-received alloy. The microstructure of the sample exposed at 270 °C for 90 h is shown in Figure 7c. In addition to a small amount of dislocations in Al matrix, there are many coarse rod-shaped particles and lath-shaped particles. It proves that the microstructure of the studied alloy is sensitive to the exposure temperature. All high-resolution transmission electron microscope (HRTEM) images were taken along <001>_Al_ direction to determine the type of particles. Figure 8 shows the HRTEM image of the studied alloy under different exposure temperature. Figure 8a displays an HRTEM image of a circular region. These precipitates are β″-Mg_2_Si particles and Guinier-preston (GP) zones, which are confirmed by the corresponding Fast Fourier Transformed (FFT) pattern (Figure 8d). Figure 8b reveals an HRTEM image of a cross-section of precipitates visible in Figure 7b; the corresponding FFT image is shown in Figure 8e, indicating that the phase present at this condition is primarily β″-Mg_2_Si, and few of β′-Mg_2_Si is also observed (not shown here). When the temperature increases to 270 °C and soaked for 90 h, many visible precipitates are observed in Figure 7c. The HRTEM (Figure 8c) and FFT image (Figure 8f) confirm that this precipitate is of Q′-AlCuMgSi phase.

## 4. Discussion

### 4.1. Evolution Mechanism of Mechanical Properties

AlSi10Mg(Cu) alloys is heat-treatable hypoeutectic aluminum alloy that can become high-strength by heat treatment. As we know, the strength of heat-treatable aluminum alloy is determined by the size and distribution of the precipitates and by coherency of the precipitates with the matrix [9]. A high density of the β″-Mg_2_Si particle (mainly precursor of β″-Mg_2_Si) and GP zones (> 2 nm in diameter) was observed in the as-received alloy (Figure 8a), indicating that the as-received alloy is in peak-age condition (T6). High concentration of vacancies and high-density of dislocations is formed after quenching [5], which can cause rapid formation of GP zones during ageing. Although the GP zones are fully coherent with Al-matrix, the elastic stress around the clusters is induced due to the difference in size between the solute (Mg, Si and Cu atom) and the Al atoms. The formation of coherent precipitates is derived from the interface energy reduced. High concentration of GP zone combined with pre-β″precipitates can hinder dislocation motion during deformation, resulting in an increase in strength [25]. The relationship between the dislocations and particulars can be expressed as [26]

(2) $$\Delta \sigma_{y}=Cf^{m}r^{n}$$

where the $\Delta \sigma_{y}$ is the yield strength increment, C, m and n are a material constant, f is the volume fraction of the shear particles, r is the radius of the shear particles. The exposure temperature of 180 °C causes the phase pre-β″ to transform to the phase β″ due to the storage at room temperature has a delaying effect upon the nucleation of pre-β″ in Al-Mg-Si alloy [27], which leads to the radius of shear particles increase. Consequently, the yield and ultimate tensile strength of the alloy increase with the extension of the exposure time. While exposure time exceeding 10 h causes particle size to exceed the critical size [5] and the number of particles to decrease, resulting in a slow decrease in alloy strength (Figure 2a). It is noted that the strength of the alloy is only slightly declined. The main reason is that the major microstructure change in this alloy is pre-β″ into β″ phase at 180 °C. The number density of β″ particle in the alloy exposed at 180 °C for 90 h, as roughly estimated from the bright-field TEM images at lower magnification, is 3.5 × 10^21^ particles/m^2^. This is consistent with results observed by in other studies [27].

However, the strength is not simply determined by the size, volume fraction, and distribution of the precipitates and the interaction of the precipitates with dislocations [28], but also determined by the coherent relationship between the precipitates and the matrix [5]. When the exposure temperature is increased to 270 °C, the tensile strength of the tested materials shows a sharply decline after 10 h and then slowly decline with further prolong exposure time. As shown in Figure 7c and Figure 8c, TEM examination shows that the particles in the alloy are converted from coherency GP zone or pre-β″ to semi-coherency Q′-AlCuMgSi phase (a small amount of β′ phase). This phenomenon was observed by Eskin [29], where the coherent β″ phase is substituted by the semi-coherent Q′ phase above 200 °C TEM research on solution-treated 6000-series alloy was also confirmed that when the alloy is over-aged, β″ precipitates are dissolved and a number of coarser precipitates formed [30]. The reasons for this may be that at the higher isothermal exposure temperature, the atoms are supposed to move over larger distances and motivation for particle transformation is enhanced [29,31]. Therefore, what is certain is that increasing the age temperature results in an increase in particle radius and a decrease in particle density [15]. The number of Q′ (In order to simplify the statistics, here we do not make a detailed distinction between Q′ and β′, but mainly Q′) is about 5 × 10^20^ particles/m^2^, which is about 10% of the alloy exposed at 180 °C for 90 h. This indicates that growth and coarsening of particle are accelerated by high temperatures. It is noteworthy that Q′ is more easily precipitated at higher temperature (270 °C) than β′ phase. This is because the copper element can move farther away at high temperature.

Compared with the as-received alloy, the number of particles dropped to 10%, while the equivalent diameter of Q′ (~21 nm in equivalent radius) was nearly seven times than that of the pre-β″ particles (~3 nm) (Figure 8a–c). The above-mentioned phenomena show that lots of clusters, as well as pre-β″ particles are substituted by the Q′ phase at the high temperature of 270 °C, which gives a lower contribution to strength [5]. The precipitation strengthening (σ) depends on the combination effect of the average particle size (*r*) and the volume fraction (*f*) [32]. Therefore, samples exposed at 270 °C show lower strength than those exposed at 180 °C as higher exposure temperature produces larger-sized precipitates (Figure 8c). Additionally, previous work on the solution-treatment Al alloy suggested that the presence of dislocation in the Al alloy has provided a substantial number of sites for the nucleation of precipitates, which is more favorable than homogeneous nucleation. The reason is perhaps that the diffusion of solute atoms can be accelerated along dislocations and the additional vacancies can be supplied to precipitates by dislocations climb. Precipitation assisted by dislocations would have enabled the nucleation and development of precipitates to occur at temperatures lower than those for homogeneous nucleation. Therefore, lots of dislocations and vacancies acted as nucleation points are consumed in the process of precipitates transformation [28], which is another reason for strength decline.

**Figure 9:**
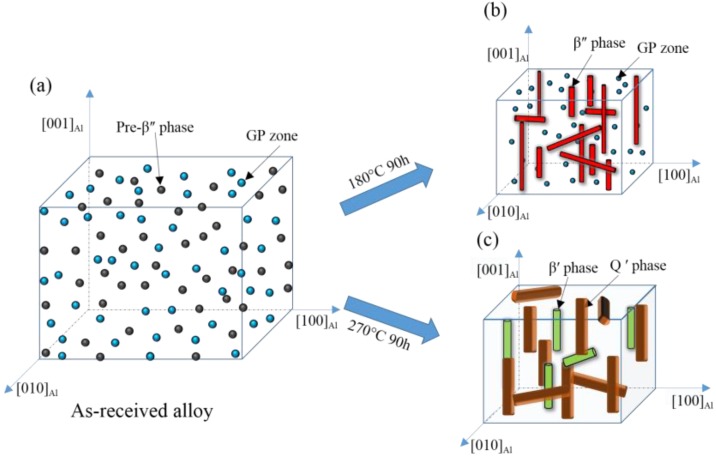
Schematic showing the microstructure of AlSi10Mg(Cu) alloy in different conditions: (**a**) as-received alloy; (**b**) exposed at 180 °C for 90 h; (**c**) exposed at 270 °C for 90 h.

As mentioned above, the mechanical properties are sensitive to the exposure temperature, which affecting the microstructure of the studied alloy. Schematic showing the microstructure of AlSi10Mg(Cu) alloy in different conditions is shown in Figure 9. As is shown in Figure 9a, the high-density of GP zone and pre-β″ particles uniformly dispersed in the Al matrix. These clusters and fine precipitates are coherent with the matrix, but the elastic stresses are induced around themselves due to the difference in size between the solute and the solvent atoms. These clusters/small particles and the stress field can hinder dislocation motion, resulting in a high strength. When the alloy is exposed to a different temperature, the precipitates are replaced by β″ particle (Figure 9b) and Q′ phase (Figure 9c), respectively, resulting in different degrees of alloy strength decline. This is a comprehensive result of phase change as well as a reduction in the density of vacancies and dislocations.

### 4.2. Evolution Mechanism of Corrosion Resistance

The presence of alloying elements Cu, Mg, Mn and Fe mainly existed in the eutectic intermetallics or precipitates have an adverse effect on the corrosion behavior of the alloy [33]. From the corrosion point of view, these compounds (such as Mg_2_Si and Q-AlCuMgSi) may lead to the formation of microgalvanic couples with the surrounding Al matrix resulting in local corrosion. So, the electrochemical behavior of the alloy is closely related to the microstructure.

According to the literature [5,13,34,35,36], it should be noted that Fe-rich intermetallics and eutectic Si phases formed during solidification are almost insoluble during the heat treatment process. For instance, Moustafa et al. [36] and Crowell et al. [37] found that phases containing Fe and eutectic Si are hard to dissolve even after long-term solution treatment (Note that the solution temperature of the cast aluminum alloy is higher than 450 °C). Therefore, it is reasonable that after exposure at 270 °C for 90 h, the effect of the Fe-rich phases and eutectic Si on corrosion resistance of the studied alloy was almost unchanged. From Figure 5 and Figure 6, it indicates that the change of corrosion parameters is mainly due to the evolution of the precipitates.

The corrosion potential represents the nucleation of metastable pits and the breakdown of the oxide layer which covers on the surface of aluminum matrix [21]. From Figure 6, it can be found that the corrosion potential increase from −0.853 V_SCE_ (as-received alloy) to −0.849 V_SCE_ (180 °C for 90 h), when the exposure temperature is increased to 270 °C, the corrosion potential further increase to −0.817 V_SCE_ (for 90 h). This indicates that the occurrence of metastable pits is more difficult when the exposure temperature is relatively higher (270 °C). Compared with the as-received alloy, the thermal exposure processing can change the density and size of precipitates in the Al matrix, resulting in the relatively high corrosion potential [21].

In order to evaluate to the corrosion resistance of the studied AlSi10Mg(Cu) alloy, the corrosion current density (*i*_corr_) and the corrosion rate (*v*) are obtained from the polarization curves and Equation (2). The corrosion rate of the studied AlSi10Mg(Cu) alloy is affected by the exposure temperature (Figure 6c), which has a similar change trend of the corrosion current (Figure 6b).

Corrosion occurs right next to the Cu/Mg rich areas, which means that the galvanic effect of the cu containing intermetallics is responsible for the dissolution of adjacent Al in these zones [10,21,33,38]. This is because there has been a large potential difference between Cu/Mg-containing compounds and the aluminum matrix. For instance, potential difference value of Mg_2_Si intermetallic particles measured in the A356 alloy is between −80 to −90 mV [6] and that of AlCuMgSi intermetallic particles measured in the A356 alloy is between −75 to −150 mV [39]. The microstructure of the specimen exposed at 180 °C for 90 h is illustrated in Figure 7b and Figure 9b. Compared with that of the as-received alloy (Figure 7a), the density of β″-Mg_2_Si phases increased significantly, resulting in more galvanic cells and the decrease of corrosion resistance. Besides, the corrosion rate increased with prolonging the exposure time. These were because the exposure temperature (180 °C) can drive the precipitates formation from the GPB zone. When the thermal exposure temperature was increased to 270 °C, the corrosion rate decline compared with that of the as-received alloy. TEM examination indicates that the pre-β″ particle is substituted by Q′-AlCuMgSi phase (Figure 7c and Figure 9c), while the number is obviously down. This is the principal reason for the increase in corrosion resistance.

## 5. Conclusions

The mechanical properties and corrosion resistance of AlSi10Mg(Cu) alloy is susceptible to the isothermal exposure parameters. Low temperature (180 °C) exposure has a great effect on the corrosion resistance. However, high temperature exposure (270 °C) has a significant impact on the tensile strength. Besides, the corresponding elongation has a similar continuing increasing tendency without exposure temperature. High-density of GP zones and pre-β″ phases are responsible for the high mechanical strength of the as-received alloy. As the studied alloy exposed at 180 °C, high-density of pre-β″ phases are partially substituted by β″ phases, which are the reason for the corrosion resistance increased. When the exposure temperature increased to 270 °C, the combined effect of coarse Q′ and few of β′ phases and the density of dislocations and vacancies dropped dramatically is responsible for the mechanical strength dramatically decrease.
